# Excess ventilation and chemosensitivity in patients with inefficient ventilation and chronic coronary syndrome or heart failure: a case–control study

**DOI:** 10.3389/fphys.2024.1509421

**Published:** 2025-01-22

**Authors:** Prisca Eser, Dominic Käesermann, Pietro Calamai, Anja Kalberer, Laura Stütz, Sarina Huber, James Duffin, Matthias Wilhelm

**Affiliations:** ^1^ Centre for Rehabilitation & Sports Medicine, Inselspital, Bern University Hospital, University of Bern, Bern, Switzerland; ^2^ Department of Anesthesia and Pain Management, University of Toronto, Toronto, ON, Canada

**Keywords:** inefficient ventilation, central chemosensitivity, cardiopulmonary exercise testing, resting ventilation, chronic coronary syndrome

## Abstract

**Background:**

In patients with chronic coronary syndromes (CCS), increased ventilation/carbon dioxide production ( $\dot{V}$ _E_/ $\dot{V}$ CO_2_) slope has been found to predict disease progression and mortality, similarly to patients with heart failure (HF); however, increased chemosensitivity, a well-established predictor for mortality in patients with HF, has rarely been assessed in patients with CCS.

**Method:**

Patients with CCS, HF with reduced ejection fraction (EF < 50%), healthy controls (45+ years), and young healthy adults (<35 years) were recruited. For patients, a $\dot{V}$ _E_/ $\dot{V}$ CO_2_ slope ≥36 was an inclusion criterion. The Duffin rebreathing method was used to determine the resting end-expiratory partial pressure of carbon dioxide (P_ET_CO_2_), ventilatory recruitment threshold (VRT), and slope (sensitivity) during a hyperoxic (150 mmHg O_2_) and hypoxic (50 mmHg O_2_) rebreathing test to determine the central and peripheral chemosensitivity.

**Results:**

In patients with CCS, HF, controls, and young healthy adults, median $\dot{V}$ _E_/ $\dot{V}$ CO_2_ slopes were 40.2, 41.3, 30.5, and 28.0, respectively. Both patient groups had similarly reduced hyperoxic VRT (at P_ET_CO_2_ 42.1 and 43.2 mmHg) compared to 46.0 and 48.8 mmHg in the control and young healthy adults. Neither hypoxic VRT nor hyper- or hypoxic slopes were significantly different in patients compared to controls. Both patient groups had lower resting P_ET_CO_2_ than controls, but only patients with HF had increased breathing frequency and rapid shallow breathing at rest.

**Conclusion:**

In patients with HF and/or CCS and excess ventilation, central chemoreflex VRT was reduced independently of the presence of HF. Low VRTs were related to resting excess ventilation in patients with CCS or HF; however, rapid shallow breathing at peak exercise was present only in patients with HF.

**Clinical trial registration number:**

NCT05057884.

## Graphical Abstract

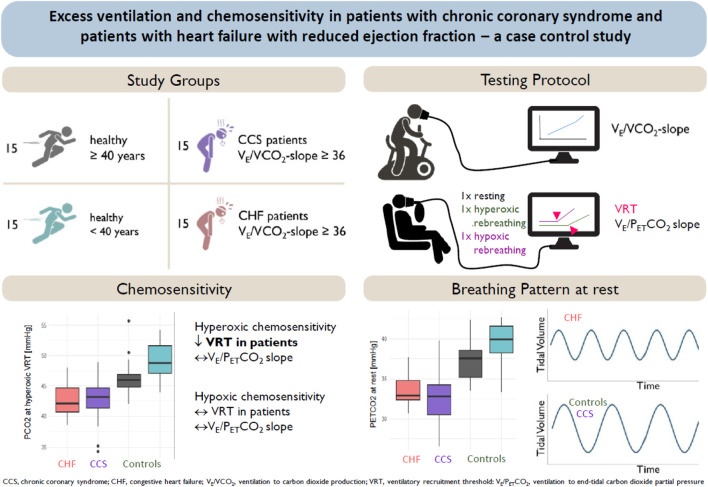

## Introduction

Chronic coronary syndromes (CCS) is the clinical presentation of coronary artery disease during stable periods, particularly those preceding or following an acute coronary syndrome (Vrints et al., 2024). CCS is the leading cause of heart failure (HF) (Ziaeian and Fonarow, 2016). HF is a clinical syndrome characterized by cardinal symptoms (e.g. breathlessness, ankle swelling, and fatigue) that may be accompanied by signs such as elevated jugular venous pressure, pulmonary crackles, and peripheral edema. It is due to a structural and/or functional abnormality of the heart, which results in elevated intra-cardiac pressures and/or inadequate cardiac output at rest and/or during exercise (McDonagh et al., 2021).

An exaggerated ventilatory response to exercise, often accompanied by early exertional dyspnea, is a hallmark in patients with chronic HF (Chua et al., 1996; Tomita et al., 2003). These observations have also been reported in patients with CCS and left ventricular dysfunction (Eser et al., 2023). Excess ventilation, often also termed ventilatory inefficiency (Neder et al., 2022), has not only been associated with reduced exercise capacity and a reduced quality of life but also with poorer prognosis in patients with HF and CCS (Ponikowski PP. et al., 2001; Arena et al., 2004; Nadruz et al., 2017; Chua et al., 1997a; Nayor et al., 2020). It is quantified by an increased $\dot{V}$ _E_/ $\dot{V}$ CO_2_ slope, arising from an excessive increase in minute ventilation (V̇_E_) with respect to carbon dioxide production ( $\dot{V}$ CO_2_) during incremental exercise in the absence of metabolic acidosis (Agostoni and Guazzi, 2017). Based on the modified alveolar equation, an increased $\dot{V}$ _E_/ $\dot{V}$ CO_2_ slope can be explained by two factors: a reduced arterial CO_2_ partial pressure (P_a_CO_2_) and/or a high fraction of the tidal volume (V_T_) that goes to dead space (V_D_) (i.e., the V_D_/V_T_-ratio) (Wang et al., 2020).

$$\frac{V_{E}}{VCO_{2}}=\frac{863}{{P_{a}CO}_{2}*\left(1-\frac{V_{D}}{V_{T}}\right)}.$$

In patients with HF, impaired cardiac function may result in lung areas that are ventilated but poorly perfused (i.e., ventilation–perfusion mismatch) with $\dot{V}$ _E_ increasing during exercise without sufficient increase in pulmonary perfusion (Weatherald et al., 2018). Furthermore, the V_D_/V_T_ ratio can be increased due to a reduced V_T_ during exercise when the diaphragm fatigues (Neder et al., 2022). Muscle fatigue in the diaphragm and/or the peripheral muscles leads to accumulating metabolites that trigger ergo reflexes, which often elicit a steep increase in the breathing frequency (Piepoli et al., 1996), resulting in a pattern of rapid shallow breathing (RSB) (Weatherald et al., 2018; Dubé et al., 2016). Increased chemosensitivity may further accelerate the abnormal ventilatory response to exercise in patients with HF (Chua et al., 1996; Ponikowski et al., 1997; Cross et al., 2020) and also in patients after acute myocardial infarction (Tomita et al., 2003).

The respiratory chemoreflexes are responsible for controlling PCO_2_, such that tissue hydrogen ion concentration ([H^+^]) is constrained within viable limits for protein function. Both the central chemo-receptors, located in the medulla, and the peripheral chemoreceptors, located in the carotid bodies, respond to [H^+^], although they are most often modeled as responding to PCO_2_ (Duffin, 2005). The peripheral chemoreceptor response to [H^+^] is modulated by partial pressure of oxygen (PO_2_), whereas the central chemoreceptor response is not (Duffin, 2005). Due to this discrepancy, by administering hypercapnia under hyperoxic conditions, the peripheral chemoreceptors can be silenced, allowing the isolated measurement of central chemoreflex (Duffin, 2005). On the other hand, administering hypercapnia under hypoxic conditions triggers both central and peripheral chemoreflexes.

Increased ventilatory drive during exercise has also been found in patients with left ventricular dysfunction but without established HF (Eser et al., 2023). Chemosensitivity, however, has not been assessed so far in patients with CCS.

Most early studies have assessed peripheral chemosensitivity to hypoxia by transient inhalation of pure nitrogen (Chua et al., 1996; Ponikowski et al., 1997; Ponikowski P. et al., 2001). However, while hypoxia may occur in some patients during sleep apnea (Giannoni et al., 2019; Giannoni et al., 2008), hypoxia does not occur during exercise and is, therefore, unlikely to be related to inefficient ventilation. More recent studies have assessed respiratory chemosensitivity in patients with HF using isocapnic steady-state rebreathing to assess peripheral chemosensitivity to decreasing partial pressures of oxygen (PO_2_) and normoxic rebreathing to assess central chemosensitivity to increasing PCO_2_ (Giannoni et al., 2008; Giannoni et al., 2009) or Read’s rebreathing method (Tomita et al., 2003; Read, 1967). However, the normoxic isocapnic steady-state method used to measure central chemosensitivity has been found to not be reproducible, while normoxic rebreathing has very good reproducibility (Mannée et al., 2018). Furthermore, steady-state methods to measure peripheral chemosensitivity apply a low PO_2_ stimulus, with PCO_2_ held eucapnic or at a fixed increase in PCO_2_ above eucapnia. However, the choice of PCO_2_ markedly influences the measured peripheral chemosensitivity, and at least two tests at differing PCO_2_ are necessary (Keir et al., 2020a). Read’s rebreathing method has been refined by Duffin by introducing a 5-min hyperventilation period to lower the arterial PCO_2._ After hyperventilation and upon rebreathing, PCO_2_ increases while $\dot{V}$ _E_ remains constant at first. At the ventilatory recruitment threshold (VRT), the $\dot{V}$ _E_ begins to increase. The measured end-tidal PCO_2_ at this point marks the VRT. It can be identified additionally to chemosensitivity, which is the increase in unit ventilation with an increasing unit of CO_2_ during isoxia at hypoxic and hyperoxic tensions (Duffin, 2011; Duffin, 2007; Mohan et al., 1999). This method has not been tested in patient populations so far, but only in a single case study (Keir et al., 2020b).

The aims of the current study were to 1) compare central and peripheral respiratory chemoreflexes in terms of VRT and sensitivity measured by the Duffin rebreathing method in patients with inefficient ventilation and CCS to those with HF and age-matched controls; 2) assess the contribution of central and peripheral chemoreflexes to ventilatory efficiency; 3) compare resting breathing patterns in these patient groups; 4) identify age-related differences in central and peripheral chemoreflexes by comparing healthy old to healthy young volunteers.

## Methods

### Study design and setting

This case–control study was conducted as a sub-study of the Breathe-HF trial (NCT05057884). Eligible patients with HF and CCS were identified and recruited during routine check-up visits at a tertiary care university cardiovascular referral center between April 2022 and April 2023. Healthy young and old volunteers were recruited by word of mouth. If they met the inclusion criteria and provided consent in writing, they were included in the study, and measurements were performed as summarized in Supplementary Figure S1. No follow-up was performed. The study was approved by the Ethics Committee of the Canton of Berne.

### Study participants

The study included four different groups of participants, two cardiac patient groups and two healthy control groups. Inclusion criteria for all groups were as follows: age 18–80 years, capability of performing a cardiopulmonary exercise test on a cycling ergometer, willingness to participate in a study of a total duration of 2 h, and provision of written informed consent. Exclusion criteria for all groups were as follows: currently smoking, non-cardiac conditions and comorbidities associated with hyperventilation such as pulmonary diseases, and pregnancy or lactation. Additional specific inclusion criteria for both patient groups were as follows: exertional dyspnea and $\dot{V}$ _E_/ $\dot{V}$ CO_2_ slope ≥36. A specific inclusion criterion for the CCS group was chronic coronary artery disease, as defined by the recent European guidelines (Vrints et al., 2024). Specific inclusion criteria for the HF group were as follows: left ventricular ejection fraction <50% and optimal guideline-directed medical therapy for >3 months. Specific exclusion criteria for patients with CCS were as follows: acute coronary syndrome in the last 3 months and signs and symptoms of heart failure assessed by history, clinical examination, and transthoracic echocardiography. A specific exclusion criterion for patients with HF was decompensation within the preceding 3 months. Inclusion age for the control group was 40–80 years and that for young healthy adults was 18–39 years. Exclusion criteria for controls and young healthy adults were as follows: past smoking and present consumption of Aspirin, statins, beta blockers, alpha blocker, blockers of the renin–angiotensin–aldosterone system, calcium-channel inhibitors, nitrates, nicorandil, ranolazine, phosphodiesterase-5-inhibitor, ivabradine, vitamin K antagonists, novel oral anticoagulants, glucocorticoids, and beta mimetics.

Participants of the patient and the control groups were recruited successively for the groups to be comparable with regard to age and sex. The young healthy adults group was included for comparison only and to assess the effect of age.

### Study procedures

#### Body composition measurement

Body composition was assessed by bioelectrical impedance with a body composition analyzer (InBody 720, best4health gmbh, Bassersdorf, Switzerland). Weight, lean muscle mass, and body fat percentage were measured. Moreover, the body mass index (BMI) was calculated.

#### Chemosensitivity measurement

Central and peripheral chemosensitivity was assessed using the rebreathing protocol according to Duffin (2011). This protocol was chosen because it can directly and reliably determine the VRT, namely, the P_ET_CO_2_ during hyper- and hypoxic conditions above which ventilation starts to increase, by establishing a PCO_2_ equilibrium between the arterial/mixed venous blood, the alveolar air, and the rebreathing bag. During hyperoxic test conditions, the peripheral chemoreflex response is reduced or eliminated, allowing quantification of the central chemoreflex only (Guluzade et al., 2023). The hypoxic test then allows measurement of the summed peripheral and central chemoreflex responses. This method also reduces the difference between end-tidal and arterial/mixed venous blood PO_2_ and PCO_2_ (Read and Leigh, 1967) and, therefore, provides the same stimulus to the central and peripheral chemoreceptors.

The prior hyperventilation of the rebreathing method by Duffin reduces PCO_2_ to below the central and peripheral chemoreceptor thresholds so that as PCO_2_ increases, the VRT can be determined. The ventilation below the VRT is a measure of the ventilation drive other than from the chemoreflexes, and since it is absent during sleep, it is therefore known as the wakefulness drive to breathe. During sleep, the VRT becomes the apnea threshold.

In contrast, other rebreathing methods extrapolate the regression line of the ventilation to P_ET_CO_2_ slope to the abscissa, which creates larger errors (Jensen et al., 2010).

Patients were previously told to abstain from caffeine on the morning of the tests. The room was dimly lit and maintained at a comfortable 22°C. The rebreathing procedure was performed using the Innocor system (COSMED Nordic ApS, Odense S, Denmark) running on a Windows XP embedded operating system on an integrated computer and a pulse oximeter (NONIN, sampling frequency 100 Hz) for O_2_ saturation. During measurements, participants wore an EU-certified breathing mask (V2 Mask, Hans Rudolph, Shawnee, USA), as used during spiro-ergometries, covering the nose and mouth. While most rebreathing procedures mentioned in literature were carried out using a mouthpiece and nose clip, Keir et al. demonstrated the feasibility of using a breathing mask for rebreathing tests (Keir et al., 2019). The breathing mask bears the advantage of allowing the participants to breathe through the nose or mouth as desired.

Once participants were fitted with the mask, they rested in a comfortable chair for 10 min. Breath-by-breath data were collected for the following parameters during resting and rebreathing: oxygen consumption relative to body weight ( $\dot{V}$ O_2_), $\dot{V}$ CO_2_, $\dot{V}$ _E_, V_T_, and breathing frequency (BF). $\dot{V}$ _E_ and V_T_ were also adjusted to body surface area (BSA). End-tidal partial pressures of oxygen (P_ET_O_2_) and carbon dioxide (P_ET_CO_2_) were measured during 100 ms of the highest O_2_ and CO_2_ values during each expiration. Instead of the proposed hyperventilation of 5 min by Duffin and colleagues (Duffin, 2011), a duration of 2 min of hyperventilation was chosen based on the findings by Boulet et al. (2016). Boulet and colleagues compared hyperventilation periods of 5, 3, and 1 min and found no difference in P_ET_CO_2_ at which the VRT occurred. They concluded that 1 min of hyperventilation prior to rebreathing was sufficient. We chose 2 min because this was a time period that was well-tolerated by patients. With longer rebreathing periods, patients complained about nausea and dizziness. After 2-min hyperventilation and a P_ET_CO_2_ decreased by at least 10 mmHg below resting measurement, participants exhaled completely, and a 3-way bi-directional valve (2100 Series, Hans Rudolph Inc.) was switched manually to connect patients with the rebreathing bag. Before starting the test, the 6-L rebreathing bag was filled to three-quarters of its volume with a gas mixture of 24% O_2_ and 6% CO_2_ and balanced by N_2_. Participants were asked to take three deep breaths to reach an equilibrium between PCO_2_ in the rebreathing bag, lungs, arterial blood, and mixed-venous blood (Duffin, 2011; Jensen et al., 1985). After this, participants were instructed to breathe calmly and comfortably. By a manually controlled flow of 100% O_2_ to the rebreathing bag, isoxia was kept at a P_ET_O_2_ of 150 mmHg. Rebreathing was terminated when P_ET_CO_2_ reached 60 mmHg or upon the participant’s request (by a previously agreed hand sign) (Keir et al., 2019).

The participant then rested for 15 min without the mask, breathing room air. (Duffin and McAvoy, 1988). During this time, the bag was washed out three times and refilled to three-quarters with a hypoxic gas mixture of 4.5% O_2_ and 6% CO_2_ and balanced by N_2_. Hypoxic rebreathing was performed following the same protocol as before, but P_ET_O_2_ was kept constant at 50 mmHg. The order of tests was kept constant for all participants to avoid the effects of hypoxia on chemosensitivity that can last several hours (Mateika et al., 1985). Parameters of the 10th minute of the resting period before the hyperoxic test (always first order) were averaged for resting values. For this period, the rapid shallow breathing index (RSBI) was also calculated as BF/V_T_ and BF/V_T_ relative to BSA.

Data analysis was performed as described by Duffin et al. (2000). Breath-by-breath P_ET_CO_2_ was plotted against time and fitted with a least-squares regression line. In order to minimize inter-breath variability, the equation for this line provided a predicted value of P_ET_CO_2_, against which $\dot{V}$ _E_ was plotted. By fitting a segmented linear regression model with a single breakpoint, the VRT after which $\dot{V}$ _E_ increased, the $\dot{V}$ _E_/P_ET_CO_2_ slope starting at the VRT was determined (Duffin et al., 2000). A subtraction of the hypoxic test slope from the hyperoxic test slope in everyone was used to estimate the peripheral chemoreflex sensitivity.

#### Cardiopulmonary exercise testing

Exercise capacity was assessed with a CPET on a cycle ergometer. Prior to the test, spirometry was used to determine forced vital capacity (FCV, l) and forced expiratory volume in one second (FEV_1_, l*min^-1^). Then, after sitting on the ergometer quietly for 3 min, blood pressure was measured two times, and the lowest measurement was recorded. A 3-min warm-up was followed by an individually set ramp. Volumes, flows, and gases were sampled continuously in an open spirometric system (Quark, Cosmed, Rome, Italy) and averaged over eight breaths. Measured variables included $\dot{V}$ O_2_, $\dot{V}$ CO_2_, $\dot{V}$ _E_, BF, V_T_, P_ET_CO_2_, heart rate (HR, beats*min^-1^), and oxygen saturation (SpO_2_, %). $\dot{V}$ O_2peak_ (ml*min^-1^*kg^-1^) was defined as the highest value of oxygen consumption averaged over 30 s. The first (VT1) and second ventilatory threshold (VT2) were identified using the Wassermann’s 9-panel plot (Marcin et al., 2020). The $\dot{V}$ _E_/ $\dot{V}$ CO_2_ slope was determined from the start of the ramp until VT2. Furthermore, the nadir of the $\dot{V}$ _E_/ $\dot{V}$ CO_2_ ratio was defined as the lowest $\dot{V}$ _E_/ $\dot{V}$ CO_2_ ratio during exercise.

### Statistical analysis

All analyses were performed by R (R Core Team, 2021, Version 4.1.0).

CCS, HF, control, and young healthy adult groups were defined as exposures. The primary outcome was central and peripheral VRT and chemosensitivity. The secondary outcomes were rapid RSBI and P_ET_CO_2_ at rest.

Baseline characteristics were tested between groups by Kruskal–Wallis tests followed by *post hoc* testing adjusted for multiple testing by Benjamini–Hochberg correction. Categorical variables were tested by Fisher’s exact tests. Associations between variables were assessed by linear regression. Statistical significance for all tests was set at a p-value <0.05.

## Results

### Study population

Each group included 15 participants (Supplementary Figure S1). Of 53 patients with CCS qualifying for the study (32.8% of screened patients had $\dot{V}$ _E_/ $\dot{V}$ CO_2_ slope ≥36), 20 could not be reached during the study period, and 18 declined participation, leaving 15 who participated in the study. Of patients with HF, 59 qualified for inclusion (29.2% of screened patients had $\dot{V}$ _E_/ $\dot{V}$ CO_2_ slope ≥36). Eighteen could not be reached during the study period, and 26 declined participation in the study. Within the HF group, eight patients were classified as having reduced (HFrEF), and seven were classified as having mildly reduced (HFmrEF) (McDonagh et al., 2021). Fifteen controls and 15 young healthy adults could be recruited. There were no significant differences between controls and the two patient groups with regard to baseline characteristics (Table 1). The only significantly different baseline characteristics between the controls and young healthy adults were age and body fat percent.

**TABLE 1:** Baseline characteristics of the two patient and two healthy groups.

	CHF patients (n = 15)	CCS patients (n = 15)	Controls (n = 15)	Young healthy (n = 15)
Age	68 (63, 71)	68 (63, 73)	68 (60, 72)	25 (24, 30)*
Female n (%)	4 (26.7)	3 (20.0)	4 (26.7)	6 (40.0)
Weight [kg]	79.9 (66.0, 84.7)	76.4 (72.0, 83.6)	76.6 (66.6, 84.3)	68.2 (63.4, 73.9)
Height [cm]	173 (164, 178)	174 (166, 178)	177 (166, 182)	173 (169, 178)
BMI [kg/m^2^]	26.2 (23.4, 28.8)	27.9 (23.1, 29.2)	25.2 (23.0, 26.1)	22.2 (21.0, 23.6)
Muscle mass [kg]^a^	36.0 (31.1, 40.5)	31.9 (30.0, 33.9)	32.1 (27.5, 36.9)	32.5 (28.6, 35.8)
Percent body fat [%]^a^	27.0 (22.1, 31.9)	30.1 (23.5, 32.5)	22.8 (19.7, 26.0)	16.9 (13.2, 18.8)^*^
Systolic BP [mmHg]	110 (100, 119)	120 (115, 135)	120 (113, 120)	117 (110, 120)
Diastolic BP [mmHg]	70 (63, 74)^*^	79 (65, 80)	80 (78, 83)	80 (76, 80)
MIP [cmH_2_O]^b^	83 (57, 98)	69 (58, 89)		
NYHA class I/II	3/12	7/8		
Presence of EOV	5 (33.3)	2 (13.3)		
LV ejection fraction [%]	40 (36, 47)	60 (57, 64)		
LAVI [mL/m^2^]	38.5 (31.4, 44.4)	29.9 (25.4, 33.3)		
LVEDVi [mL/m^2^]	68.0 (54.5, 98.2)	46.4 (45.1, 53.9)		
History of ACS (%)	8 (53,3)	10 (66.7)		
History of PCI (%)	9 (60.0)	11 (73.3)		
Cardiac implantable electric device (%)	9 (60.0)	1 (6.7)		
*Medication*				
ACEi/AT2 (%)	3 (20.0)	13 (86.7)		
ARNI (%)	9 (60.0)	0 (0.0)		
MRA (%)	8 (53.3)	0 (0.0)		
SGLT2i (%)	14 (93.3)	4 (26.7)		
Beta blocker (%)	12 (80.0)	12 (80.0)		
Carvedilol (%)	0 (0)	0 (0)		

Data are indicated as median (1^st^, 3^rd^ quartiles) or number of subjects (%).

^*^ Adjusted p-value <0.05 of *post hoc* Kruskal–Wallis tests between old control subjects and other groups.

^a^ Data missing from one CCS and six CHF patients due to the inability to conduct body composition measurement because of cardiac implantable electric devices (CIEDs).

^b^ Data are available only from nine CHF and 11 CCS patients.

CHF, chronic heart failure; CCS, acute/chronic coronary syndrome; BMI, body mass index; BP, blood pressure; MIP, maximal inspiratory pressure; NYHA class, New York Heart Association class; EOV, exercise oscillatory ventilation; LV, left ventricular; LAVI, left atrial volume index; LVEDVi, left ventricle end-diastolic volume index; ACS, acute coronary syndrome; PCI., percutaneous coronary intervention; ACEi, Angiotensin-converting-enzyme inhibitors; AT2, angiotensin II, type 2 receptor agonist; ARNI, angiotensin receptor–neprilysin inhibitor; MRA, mineralocorticoid receptor antagonist; SGLT2i, mineralocorticoid receptor antagonist inhibitor.

### Results of chemosensitivity tests

There was mask leakage in one control during the hyperoxic test and in another control in the hypoxic test, leaving the results of only 14 subjects in this group for data analysis. During the hypoxic test, there was mask leakage in two patients with HF and four patients with CCS. Furthermore, one patient with HF and two patients with CCS stopped the hypoxic tests after 3–5 breaths, which did not allow the determination of VRT and slope. Therefore, only 12 patients were included for data analysis of the hypoxic test. A typical example of the sampled data of the two tests in one patient with HF and one age- and sex-matched control is shown in Supplementary Figure S2.

**FIGURE 1:**
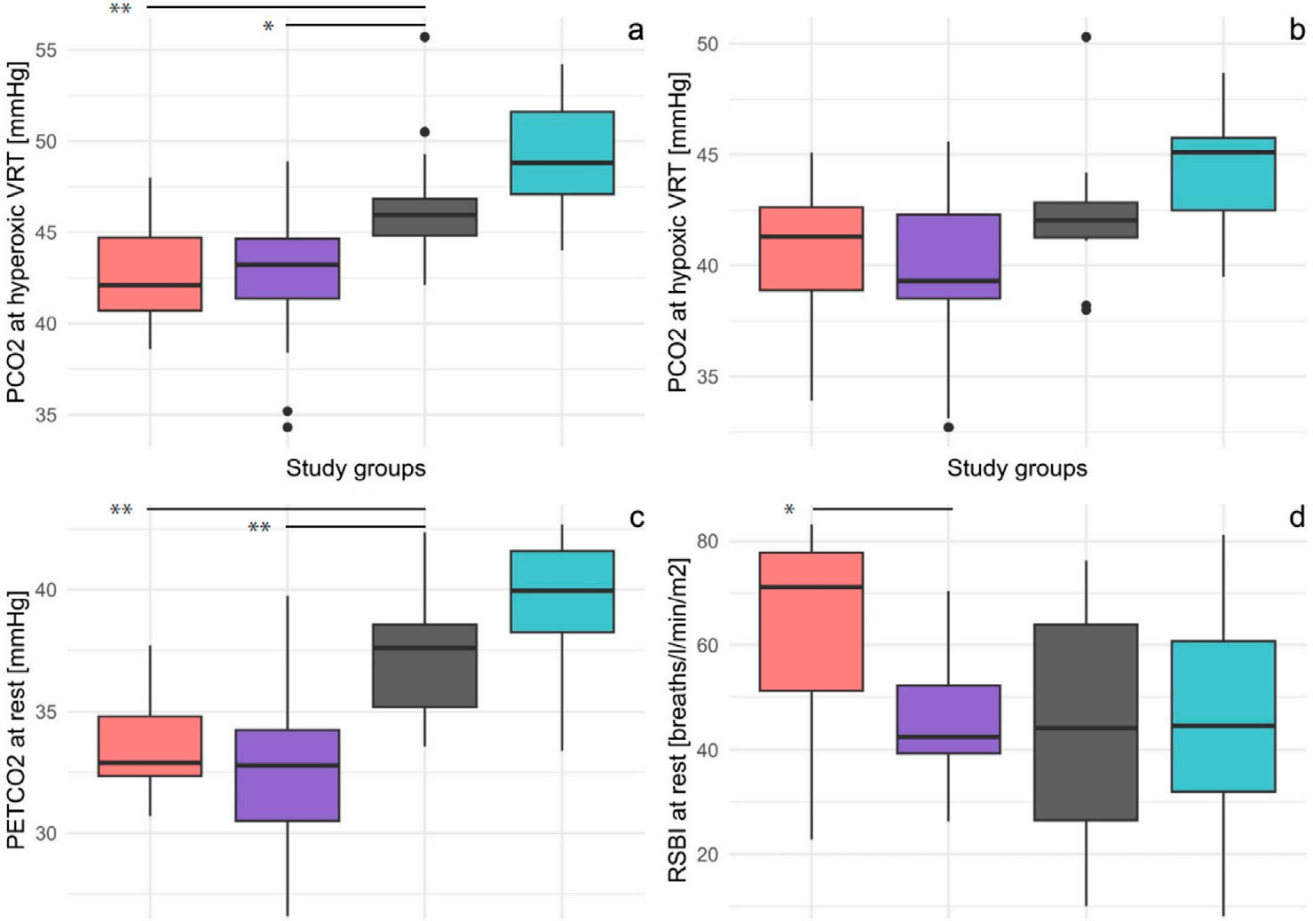
Boxplots of the four study groups showing P_ET_CO_2_ at hyperoxic VRT **(A)**, P_ET_CO_2_ at hypoxic VRT **(B)**, P_ET_CO_2_ at rest **(C)**, and RSBI **(D)**. VRTs were determined from rebreathing and RSBI and P_ET_CO_2_ from resting measurement before rebreathing. VRT of the hyperoxic test was significant, and VRT of hypoxic test by trend reduced in both patient groups compared to that in controls. P_ET_CO_2_ was significantly reduced in both patient groups compared to controls. RSBI was increased compared to controls only in patients with HF. * p-value<0.05, ** p-value<0.01; CHF, chronic heart failure; CCS, chronic coronary syndrome; PCO_2_, end-tidal carbon dioxide pressure; VRT, ventilatory response threshold; RSBI, rapid shallow breathing index; P_ET_CO_2_, end-tidal carbon dioxide pressure.

The HF and CCS patient groups had significantly reduced median hyperoxic test VRTs compared to controls (p = 0.004 and 0.01, respectively, Figure 1A; Table 2), but the hypoxic test VRTs did not differ (Figure 1B; Table 2). Young healthy adults had higher median VRTs than controls for both hyper- and hypoxic tests, which were not quite significant (p = 0.06 and p = 0.07, respectively).

**TABLE 2:** Parameters measured with hyperoxic and hypoxic rebreathing.

	VRT pCO_2_ [mmHg]	Slope [l⋅min^-1^⋅mmHg^-1^]	Peripheral slope [l⋅min^-1^⋅mmHg^-1^]
*Hyperoxic trial [pO*_*2*_ *150 mmHg]*			
CHF patients (n = 15)	42.1 (40.7, 44.7)^a^	2.90 (2.05, 4.55)	
CCS patients (n = 15)	43.2 (41.3, 44.7)^b^	2.60 (2.05, 2.95)	
Controls (n = 14)	46.0 (44.8, 46.9)	2.45 (1.40, 3.27)	
Young healthy adults (n = 15)	48.8 (47.1, 51.6)	2.10 (1.60, 3.45)	
*Hypoxic trial [pO*_*2*_ *50 mmHg]*			
CHF patients (n = 12)	41.3 (38.9, 42.6)	5.35 (2.78, 9.15)	1.55 (0.78, 2.65)
CCS patients (n = 9)	39.3 (38.5, 42.3)	4.90 (4.00, 7.70)	3.10 (2.60, 3.20)
Controls (n = 14)	42.1 (41.3, 42.9)	4.70 (3.10, 5.40)	1.90 (1.50, 2.60)
Young healthy adults (n = 15)	45.1 (42.5, 45.8)	4.70 (3.75, 6.75)	2.10 (1.50, 3.15)

^b^ Dunn *post hoc* testing with Benjamini–Hochberg correction p < 0.05 against old controls.

^a^ Dunn *post hoc* testing with Benjamini–Hochberg correction p < 0.01 against old controls.

VRT, ventilatory threshold; VE, ventilation; pCO_2_, partial pressure of carbon dioxide; pO_2_, partial pressure of oxygen.

In all groups, hypoxic test slopes were significantly higher than hyperoxic test slopes (all p < 0.004, Table 2). However, hyperoxic and hypoxic test slopes (sensitivity; $\dot{V}$ _E_ vs. P_ET_CO_2_) were not significantly different between the patient groups and controls (Table 2). Hyperoxic and hypoxic slopes were not different between young healthy adults and controls.

**FIGURE 2:**
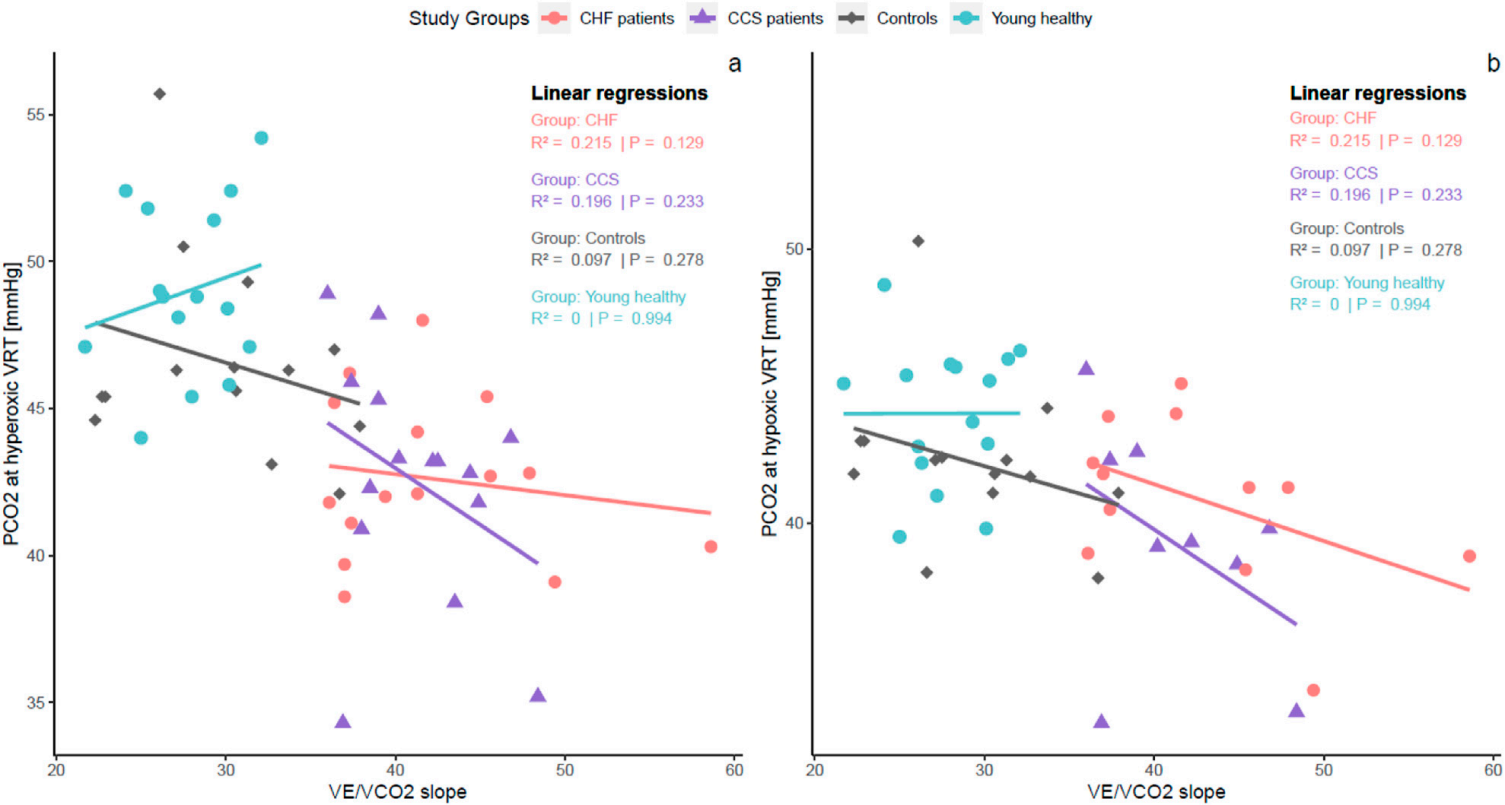
Within-group linear regressions between $\dot{V}$ _E_/ $\dot{V}$ CO_2_ slope of CPET and PCO_2_ at hyper **(A)** and hypoxic **(B)** VRTs of the chemosensitivity measurements. Within-group relationships between $\dot{V}$ _E_/ $\dot{V}$ CO_2_ slope and VRTs were absent or weak, indicating that other mechanisms than chemosensitivity are involved with inefficient ventilation during exercise. On the other hand, high $\dot{V}$ _E_/ $\dot{V}$ CO_2_ slopes were related to low levels of maximal PCO_2_ during exercise. CHF, chronic heart failure; CCS, chronic coronary syndrome; $\dot{V}$ _E_, ventilation; $\dot{V}$ CO_2_, carbon dioxide production; PCO_2_, end-tidal carbon dioxide pressure; VRT, ventilatory response threshold; CPET, cardiopulmonary exercise test.

Linear inverse relationships between $\dot{V}$ _E_/ $\dot{V}$ CO_2_ slopes and hyperoxic and hypoxic VRT were only significant for the pooled sample (*r* ^2^ = 0.35 and *r* ^2^ = 0.24; both p < 0.0001) but not within groups (Figures 2A,B). There were no linear relationships between $\dot{V}$ _E_/ $\dot{V}$ CO_2_ slopes and hyperoxic and hypoxic test slopes of the rebreathing tests within groups or in the pooled sample. However, there was a significant linear inverse association between $\dot{V}$ _E_/ $\dot{V}$ CO_2_ slopes and the maximal P_ET_CO_2_ reached during the CPET. This finding was true for both within groups (all p < 0.02) and for the pooled sample (*r* ^2^ = 0.79, p < 0.0001).

**FIGURE 3:**
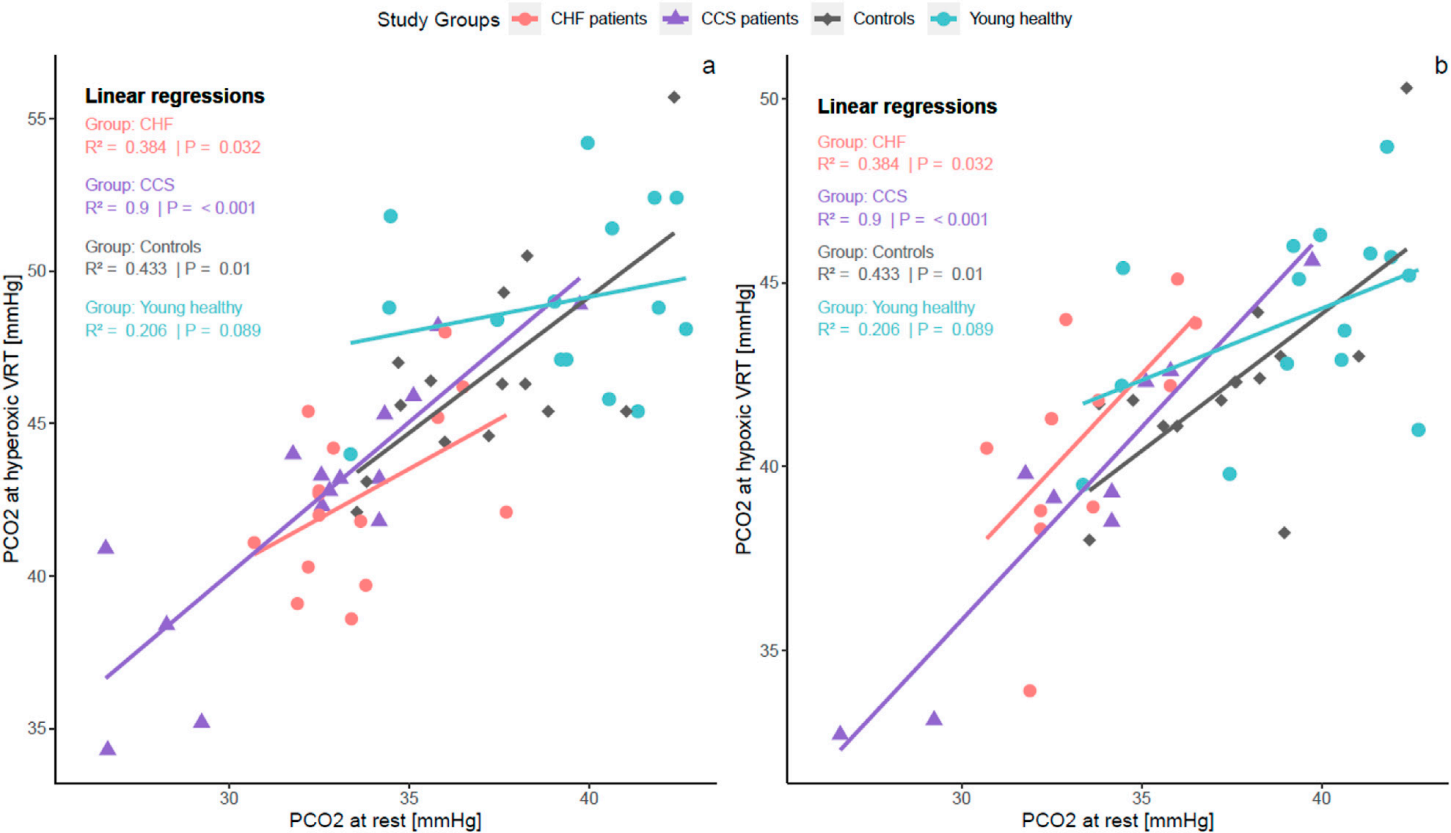
Within-group linear regressions between resting P_ET_CO_2_ and hyper **(A)** and hypoxic VRT **(B)**. There were strong positive within-group relationships in all groups except young healthy adults. These relationships indicate that a reduced VRT is connected to hyperventilation at rest. PCO_2_, end-tidal carbon dioxide pressure; VRT, ventilatory response threshold.

There was a significant positive linear correlation between hyperoxic and hypoxic VRT in the pooled sample (*r* ^2^ = 0.68, p < 0.0001) and within each group (all p ≤ 0.05; Supplementary Figure S3A). Correlations between hyper- and hypoxic slopes were significant and positive for the pooled sample (*r* ^2^ = 0.24; p < 0.0001) and within all groups (p < 0.006) except the CCS group (p = 0.201; Supplementary Figure S3B). P_ET_CO_2_ at rest was positively related to hyperoxic and hypoxic test VRTs within all groups except for the hyperoxic VRT of the young healthy adults group (Figures 3A,B).

### Results of resting measurements and cardiopulmonary exercise tests

Resting parameters of the two patient groups were comparable to those of the control subjects except for P_ET_CO_2_, which was lower in both patient groups (Figure 1C), and BF and RSBI (Figure 1D), which were higher in the HF group compared to that in the controls and patients with CCS (Supplementary Table S1). Based on the inclusion criteria for both patient groups, they had significantly higher $\dot{V}$ _E_/ $\dot{V}$ CO_2_ slopes, nadir $\dot{V}$ _E_/ $\dot{V}$ CO_2_, and lower maximal P_ET_CO_2_ (Supplementary Table S1). At peak exercise, power, VO_2,_ absolute and relative (to body surface) $\dot{V}$ _E_, P_ET_CO_2_, and HR were lower than those in the control subjects (Supplementary Table S1). Patients with HF had higher BF and RSBI than patients with CCS (p < 0.05 for BF and RSBI relative to BSA; p = 0.05 for RSBI).

## Discussion

### General

The present study selected patients with chronic HFrEF/HFmrEF or CCS who presented with exercise excess ventilation and compared their central and peripheral chemosensitivity and resting breathing pattern to that in age-matched controls and young healthy subjects. This is the first study that included two cohorts of well-phenotyped patients with HF (with reduced ejection fraction) and CCS and simultaneously assessed breathing efficiency at rest with standardized tests as well as at exercise with CPET-based measurements. Furthermore, the inclusion of both sexes and a young healthy group allowed a direct comparison with healthy subjects without ventilatory inefficiency and the effect of age on the measured parameters.

Key findings of the study were: 1) patients with CCS and HF had lower hyperoxic rebreathing test VRTs compared to control and young healthy subjects. 2) Central and peripheral chemosensitivity quantified by $\dot{V}$ _E_/P_ET_CO_2_ slopes were not different between groups. 3) Both patient groups had excess ventilation at rest with lower P_ET_CO_2_ compared to the controls, which correlated with the decreased hyperoxic and hypoxic rebreathing test VRTs. 4) Patients with HF had a rapid shallow breathing pattern and higher BF and RSBI at rest compared to patients with CCS and controls. This is the first study to show that excess ventilation, which has been well-investigated with exercise and whose negative correlation with poor outcome has been well-investigated in patients with HF, is also present at rest, and excess ventilation at rest is also present in patients with CCS. This suggests that excess ventilation in these patients may, at least in part, be caused by factors other than congestion of the lung due to reduced cardiac function and is not only elicited by exercise.

### Chemoreflex ventilatory recruitment threshold

A lower VRT in patients with HF has previously been found in patients with univentricular heart using both transient hypoxia and Read rebreathing tests (Chua et al., 1997b). Reduced VRT reflects excess ventilation (e.g. altitude hypoxia (Frost et al., 2024)), which in this case likely results from high or normal anion gap metabolic acidosis and reduced bicarbonate (Fenves and Emmett, 2021; Ren and Robbins, 1999). The VRT of the hyperoxic test depends on the buffering capacity of the brain fluid, and the VRT of the hypoxic tests depends on the buffering capacity of the blood (Duffin, 2005). PCO_2_ of the brain fluid and blood are correlated during rebreathing (Carr et al., 2023). This is likely the reason for the close association between hyper- and hypoxic VRTs (Supplementary Figure S3). Electrolyte derangement such as by hyponatremia, hypocalcemia, hypokalemia, hypomagnesemia, and hypophosphatemia are common in patients with HF, as documented by several studies (Milionis et al., 2002; Urso et al., 2015; Dei Cas et al., 1995). Importantly, central chemosensitivity has been found to be significantly reduced in patients with HF after intravenous iron infusion (Caravita et al., 2022). The fact that patients with CCS and HF had comparable values of reduced ventilatory efficiency, reduced VRT, and reduced P_ET_CO_2_ at rest suggests that they had the same degree of excess ventilation, although with a different breathing pattern where only patients with HF developed a rapid shallow breathing pattern. This indicates that the HF-related congestion of the lung is likely responsible for the increased $\dot{V}$ _E_ to be achieved by increasing breathing frequency rather than greater tidal volume (Dubé et al., 2016), but it also indicates that low cardiac output may not be responsible for increased ventilatory drive. Hyperoxic and hypoxic VRTs and, hence, possibly electrolyte status correlated positively with resting P_ET_CO_2_; however, VRTs correlated poorly with ventilatory drive during exercise ( $\dot{V}$ _E_/ $\dot{V}$ CO_2_ slope), indicating other mechanisms (such as sympathetic hyperactivation) that contribute to ventilatory drive during exercise.

Furthermore, hyperoxic and hypoxic rebreathing test $\dot{V}$ _E_/P_ET_CO_2_ slopes did not correlate with $\dot{V}$ _E_/ $\dot{V}$ CO_2_ slopes. This finding is in contrast to that of a study by Tomita and colleagues, who found a significant correlation between $\dot{V}$ _E_/ $\dot{V}$ CO_2_ slope and $\dot{V}$ _E_/P_ET_CO_2_ slopes during hyperoxic Read rebreathing (Tomita et al., 2003). Their hyperoxic testing procedure differed from ours, in that PO_2_ decreased during the test, while it was kept isoxic in ours. There are also other studies that found some association between $\dot{V}$ _E_/ $\dot{V}$ CO_2_ slope and peripheral chemosensitivity (Chua et al., 1996) and central chemosensitivity (Chua et al., 1996; Giannoni et al., 2008). The weak contribution of chemosensitivity to exercise ventilatory efficiency suggests that other factors are important. A likely culprit for an elevated $\dot{V}$ _E_/ $\dot{V}$ CO_2_ slope in patients with HF or CCS may be the ergoreflex (Piepoli et al., 1996; Aimo et al., 2021; Piepoli et al., 1999). Scott and colleagues found a significant positive relationship between $\dot{V}$ _E_/ $\dot{V}$ CO_2_ slope and increase in ventilation above rest at 2 min after hand grip exercise and post-exercise regional circulatory occlusion, a test quantifying metaboreflex, in 15 patients with CHF and eight healthy controls (Scott et al., 2002). Our results indicate that the direct contribution of chemosensitivity to exercise ventilatory efficiency is minimal in both patients with HF or CCS.

The age-related decrease in VRT for both hyperoxic and hypoxic rebreathing tests observed in our control and young healthy groups corresponds to findings of earlier studies (García-Río et al., 2007). Garcia-Rio and colleagues found the VRTs of both hyperoxic hypercapnic and isocapnic hypoxic stimulation in elderly subjects to be at a lower P_ET_CO_2_ than in young healthy subjects (García-Río et al., 2007). However, they found that the hyperoxic hypercapnic VRT increased again after age 75.

### Chemoreflex sensitivity

Our findings of comparable V_E_/P_ET_CO_2_ slopes of the hyper- and hypoxic rebreathing between our patient and healthy groups are in contrast to those of some previous studies that have reported increased central chemosensitivity to hypercapnia (Giannoni et al., 2008; Giannoni et al., 2009; Giannoni et al., 2022; Narkiewicz et al., 1999) and peripheral chemosensitivity to hypoxia in heart failure (Ponikowski et al., 1997; Giannoni et al., 2008; Giannoni et al., 2009; Giannoni et al., 2022; Chua et al., 1997c). Tomita et al. (2003) found increased chemosensitivity in patients with acute myocardial infarction using Read hyperoxic rebreathing tests, and peripheral chemosensitivity measured using the Duffin isoxic rebreathing method has been found to be associated with cardiovascular risk in a Chinese population (Dai et al., 2024).

We found no differences between hyperoxic and hypoxic slopes (sensitivity) between young healthy adults and controls, which is in agreement with previous findings. Increased chemosensitivity with increasing age was found during sleep (Chowdhuri et al., 2015), but another study found no differences between healthy younger and older men with transient hypoxia by rebreathing of pure nitrogen by another study (Paleczny et al., 2014).

### Resting P_ET_CO_2_

Our study is the first to show that in patients with inefficient ventilation, resting P_ET_CO_2_ is lower than that in controls regardless of left ventricular ejection fraction. While excess ventilation with exercise has been well-studied, resting excess ventilation has rarely been investigated. Resting P_a_CO_2_ <31 mmHg has been found to be associated with increased all-cause mortality (Kato et al., 2021). We have recently shown that increased resting breathing frequency was associated with major adverse events in patients with left ventricular dysfunction (Eser et al., 2023). The association of a lower resting P_a_CO_2_ with VRT or chemosensitivity has not been investigated, although in patients with HF, it has been found that Cheyenne Stokes respiration was associated with a lower resting P_a_CO_2_ (Naughton et al., 1993). It has been suggested that low P_a_CO_2_ in patients with HF may be a respiratory manifestation of elevated left ventricular filling pressures (Lorenzi-Filho et al., 2002). However, since we found the same relationships between VRT and resting P_ET_CO_2_ in all groups (with healthy control groups and patients with CCS not having elevated left ventricular filling pressures), our data suggest that there may be a neurological or hormonal rather than a circulatory cause for the resting excess ventilation in patients with HF and CCS.

### Limitations

As in a recent validation study (Jensen et al., 2010), we found the Duffin hyperoxic and hypoxic rebreathing tests to be feasible in healthy controls but less feasible in patients with CCS or HF. One problem, particularly during the hypoxic test, was that after hyperventilation and the subsequent three breaths for equilibration, many patients took a break from breathing for a few seconds to recover from hyperventilation. When breathing resumed, they had already surpassed their VRT and stopped the test after only a few more breaths. The reduction in breaths reduced the quality of VRT and $\dot{V}$ _E_/P_ET_CO_2_ slope determinations. Mask leakage was an additional problem in our study; however, sealing the lips around a mouthpiece can also be challenging. The consequent exclusion of some hypoxic rebreathing tests may have led to underpowering of the hypoxic test. Furthermore, since we only included patients with $\dot{V}$ _E_/ $\dot{V}$ CO_2_ slopes ≥36, we cannot extrapolate our findings of similarly altered chemoreflexes and reduced P_ET_CO_2_ in patients with CCS and HF to patients with only mildly increased ventilatory inefficiency. Last but not least, we only measured the ventilatory response to increasing values of P_ET_CO_2_. Due to the absence of simultaneous blood gas analysis, we assumed that P_ET_CO_2_ reflected arterial PCO_2_, an assumption supported by recent experimental evidence (Carr et al., 2023).

### Future implications

Measurements of chemoreflexes are time-consuming, technically challenging, and uncomfortable for patients. The measurement of resting ventilation, however, is relatively easy in comparison, with the only requirement that patients need to sit calmly for 5 min and the face masks need to be tight. The close relationship between resting P_ET_CO_2_ and hyper- and hypoxic VRTs indicates a deficit in buffering capacity of the blood in patients with HF or CCS and low P_ET_CO_2_. Our previous findings from a study on patients with left ventricular dysfunction indicating higher incidence of major adverse cardiac events in patients with lower P_ET_CO_2_ (Eser et al., 2023) indicate that resting ventilation may be an under-recognized and valuable prognostic marker.

## Conclusion

The present study shows that 1) there was no difference between patients with HF and patients with CCS regarding VRT and chemosensitivity, and in both patient groups, VRT was lower compared to that in controls, while chemosensitivity was only slightly and insignificantly heightened in patients; 2) neither VRT nor chemosensitivity was associated with the degree of inefficient ventilation within any of the groups; 3) both patient groups had excess ventilation at rest; 4) neither VRT nor chemosensitivity was associated with age. The absence of differences regarding VRT and chemosensitivity between the two patient groups challenges the notion that inefficient ventilation and excessive ventilatory drive are due to circulatory weakness. On the other hand, the presence of rapid shallow breathing at rest found in patients with HF only was likely due to lung congestion.

## Data Availability

The raw data supporting the conclusions of this article will be made available by the authors, without undue reservation.

## Abbreviations

BMI, body mass index; BF, breathing frequency; CCS, chronic coronary syndromes; CO_2_, carbon dioxide; CPET, cardiopulmonary exercise test; HF, heart failure; HFpEF, heart failure with preserved ejection fraction; HFmrEF, heart failure with mildly reduced ejection fraction; HFrEF, heart failure with reduced ejection fraction; HR, heart rate; O_2_, oxygen; P_a_CO_2_, arterial partial pressure of carbon dioxide; P_a_O_2_, arterial partial pressure of oxygen; P_ET_CO_2_, end-tidal partial pressure of carbon dioxide; P_ET_O_2_, end-tidal partial pressure of oxygen; RPE, rate of perceived exertion; RSBI, rapid shallow breathing index; SpO_2_, oxygen saturation; V_D_, pulmonary dead space; $\dot{V}$ _E_, ventilation; $\dot{V}$ CO_2_, carbon dioxide production; $\dot{V}$ O_2_, oxygen production; V_T_, tidal volume; VT1, first ventilatory threshold; VT2, second ventilatory threshold; VRT, ventilatory recruitment threshold.
