# Supplementary figures and images for: Excess ventilation and chemosensitivity in patients with inefficient ventilation and chronic coronary syndrome or heart failure: a case–control study
Source: Front Physiol. 2025 Jan 22;15:1509421. doi: 10.3389/fphys.2024.1509421 (PMC11794504; doi:10.3389/fphys.2024.1509421)

Supplement Figure 2

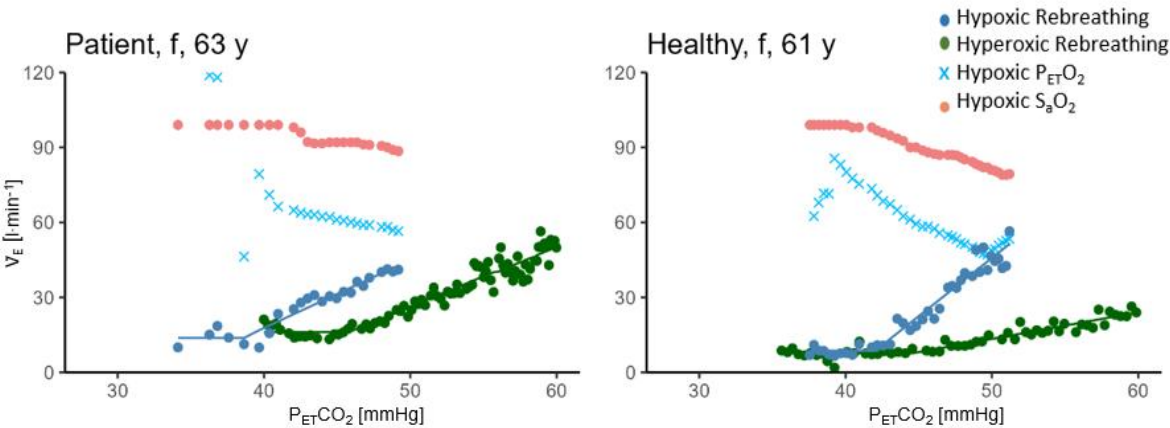

Supplement: Supplementary file 1 [file DataSheet2.pdf]

Study Groups CHF patients CCS patients Controls Young healthy

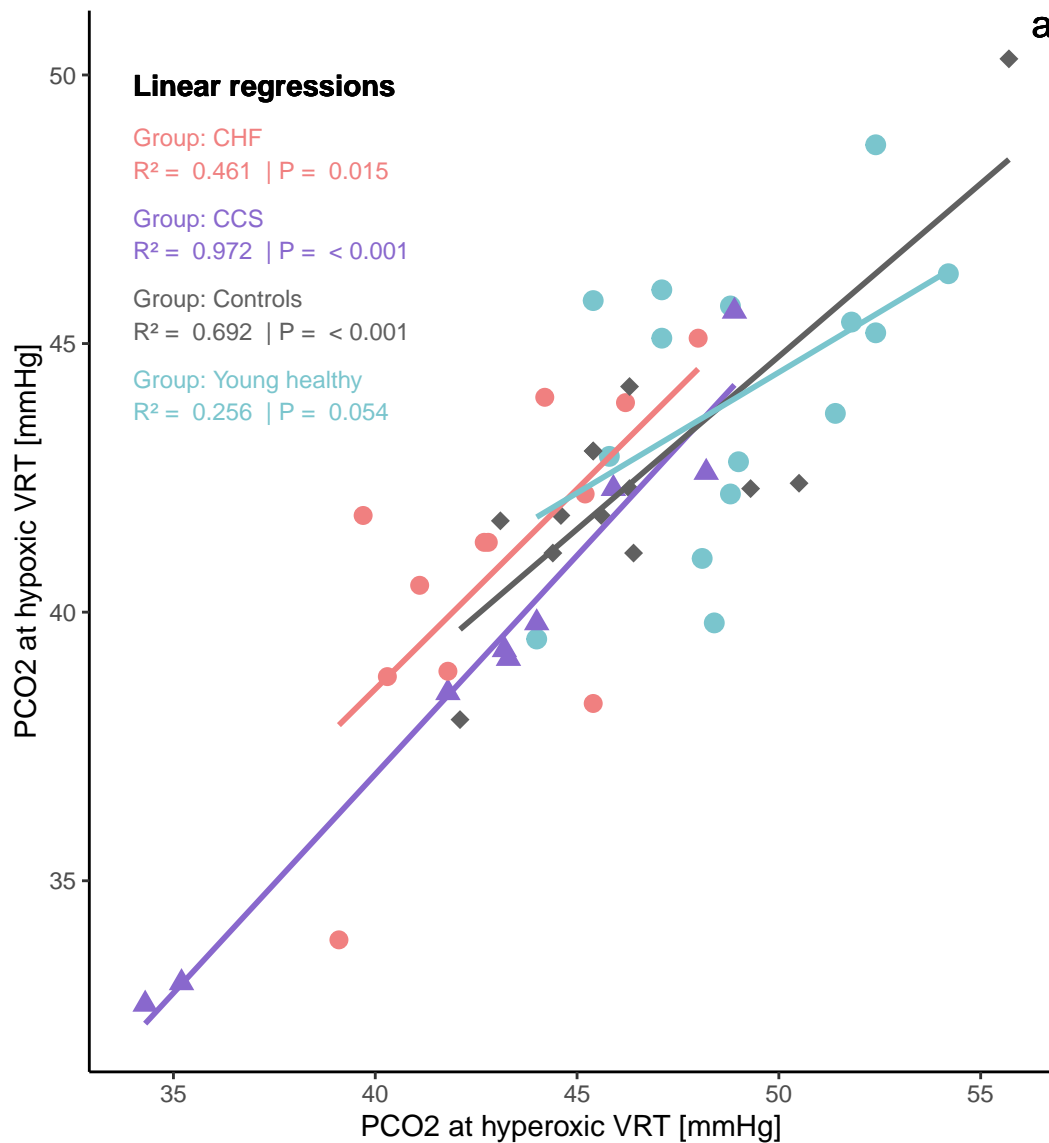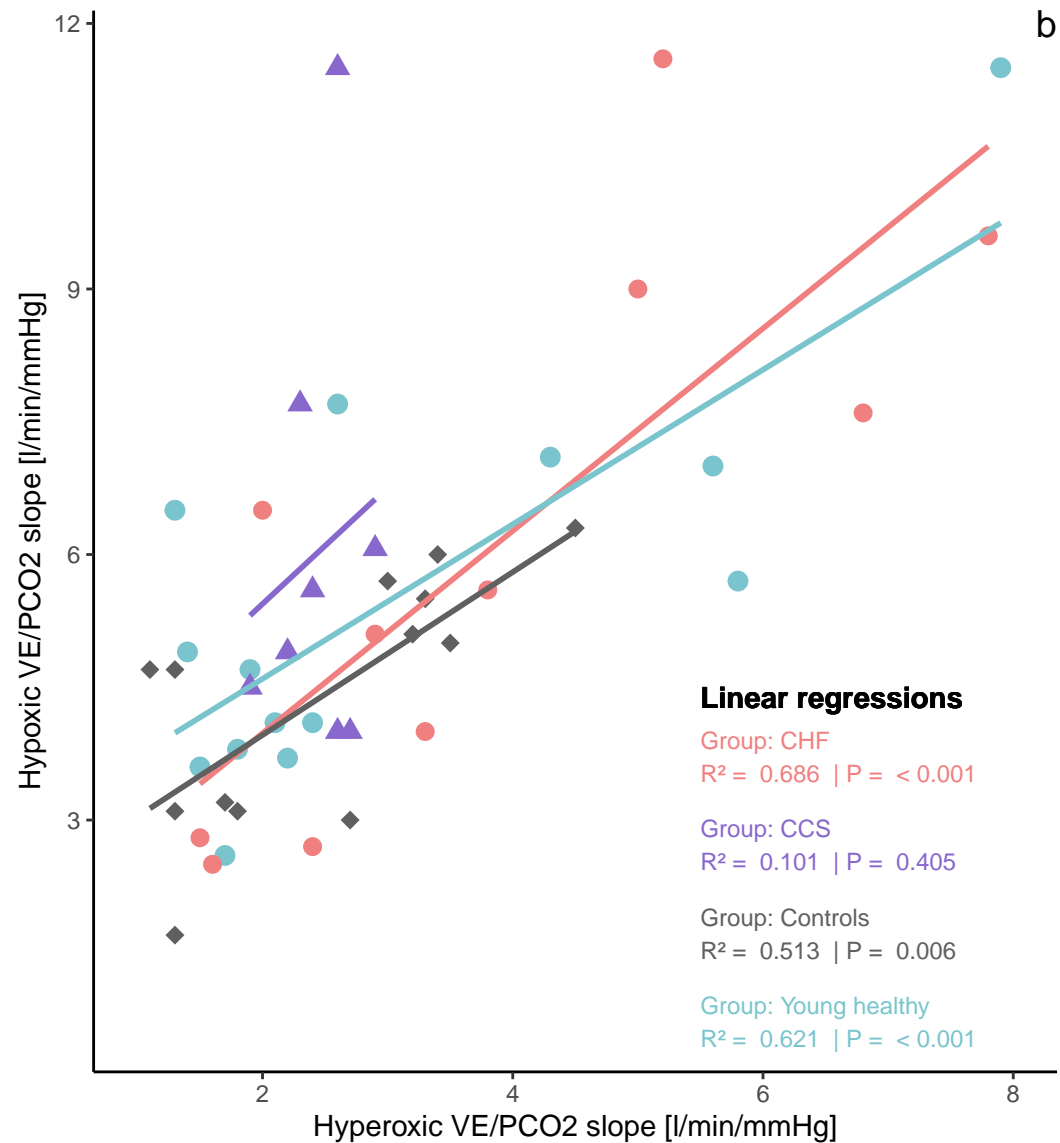

Supplement: Supplementary file 3 [file DataSheet3.pdf]

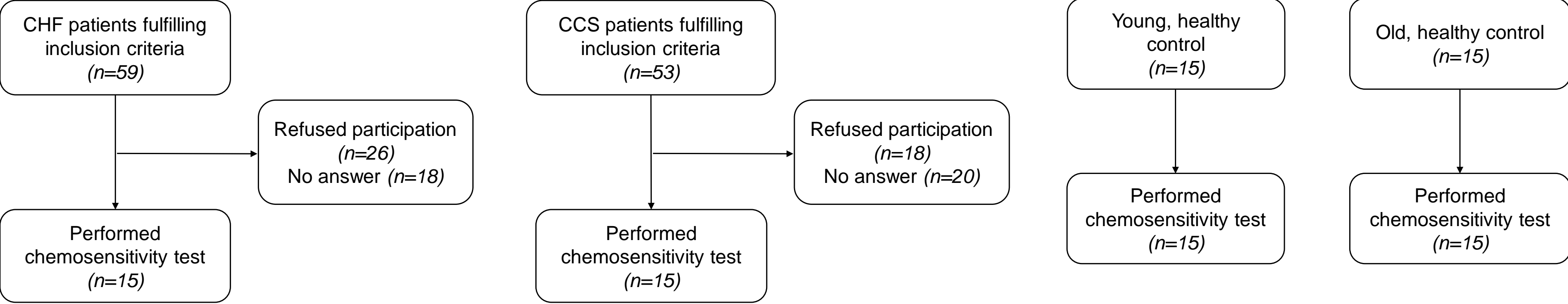

Supplement: Supplementary file 4 [file DataSheet1.pdf]
