# Supplementary material for: Excess ventilation and chemosensitivity in patients with inefficient ventilation and chronic coronary syndrome or heart failure: a case–control study
Source: Front Physiol. 2025 Jan 22;15:1509421. doi: 10.3389/fphys.2024.1509421 (PMC11794504; doi:10.3389/fphys.2024.1509421)
Supplement: Supplementary file 2 [file Table1.docx]

Supplement Table 1: Parameters from last minute of 10-min resting phase and spirometry, cardiopulmonary exercise testing during and at peak exercise.

|  | **CHF patients**  **(n = 15)** | **CCS patients**  **(n = 15)** | **Controls**  **(n = 15)** | **Young healthy**  **(n = 15)** |
| --- | --- | --- | --- | --- |
| ***Resting*** |  |  |  |  |
| Ventilation [l∙min^-1^] | 14.3 (12.1, 16.7) | 14.0 (12.3, 14.9) | 12.5 (10.3, 13.3) | 12.4 (10.5, 15.0) |
| Ventilation [l∙m^-2^ min^-1^] | 4.62 (4.10, 5.40) | 4.87 (4.62, 5.06) | 3.98 (3.67, 4.22) | 4.14 (3.72, 4.79) |
| Breathing frequency [min^-1^] | **17.5 (16.8, 19.2)^†^** | 14.8 (13.4, 15.4) | 13.0 (10.2, 16.2) | 13.0 (11.0, 17.0) |
| Tidal volume [l] | 0.77 (0.71, 1.01) | 0.95 (0.81, 1.06) | 0.92 (0.84, 1.07) | 0.90 (0.84, 1.14) |
| Tidal volume [l∙m^-2^] | 0.27 (0.25, 0.33) | 0.34 (0.27, 0.36) | 0.31 (0.27, 0.36) | 0.30 (0.28, 0.36) |
| RSBI [breaths/min/l] | 20.8 (17.1, 27.2) | 15.4 (12.9, 18.3) | 13.8 (10.1, 19.5) | 15.5 (10.9, 19.4) |
| RSBI [breaths/min/l/m^2^] | **71.1 (51.3)*** | 42.5 (39.3, 52.3) | 44.3 (26.6, 63.9) | 44.6 (32.0, 60.7) |
| P_ET_CO_2_ [mmHg] | 32.9 (32.4, 34.8)^†^ | 32.8 (30.5, 34.3)^†^ | 37.6 (35.2, 38.6) | 40.0 (38.2, 41.6) |
| Heart rate [bpm] | 62 (54, 67) | 66 (60, 71) | 66 (59, 71) | 65 (61, 76) |
| FVC [l] | 3.62 (3.14, 4.14) | 3.97 (3.57, 4.80) | 4.43 (3.96, 4.96) | 5.42 (4.51, 5.66) |
| FEV_1_ [l*min^-1^] | 2.77 (2.47, 3.24) | 2.96 (2.89, 3.41) | 3.35 (2.85, 3.96) | 4.20 (3.76, 4.57)* |
| ***During ramp exercise*** |  |  |  |  |
| V̇_E_/V̇CO_2_-slope | 41.3 (37.2, 45.5)^‡^ | 40.2 (38.3, 44.0)^‡^ | 30.5 (26.4, 33.2) | 28.0 (25.8, 30.3) |
| Nadir V̇_E_/V̇CO_2_ | 36.9 (35.3, 39.2)^‡^ | 38.0 (35.0, 39.1)^‡^ | 28.5 (25.7, 30.5) | 26.3 (24.7, 27.3) |
| P_ET_CO_2_ max. [mmHg] | 31.0 (29.5, 34.0)^‡^ | 32.0 (30.5, 34.0)^‡^ | 40.0 (38.5, 44.5) | 42.0 (40.5, 45.0) |
| ***At peak exercise*** |  |  |  |  |
| Power [watt] | 104 (71, 125)^‡^ | 142 (121, 180)* | 210 (193, 235) | 299 (258, 343) |
| Power [watt∙kg^-1^] | 1.42 (0.94, 1.70)^‡^ | 1.81 (1.58, 2.08)* | 2.80 (2.58, 3.79) | 4.59 (3.89, 4.96)* |
| V̇O_2_ [ml∙kg^-1^∙min^-1^] | 16.3 (12.9, 21.0)^‡^ | 20.9 (19.3, 23.0)^†^ | 32.6 (27.5, 37.8) | 46.8 (43.4, 52.3) |
| V̇O_2_ of predicted [%] | 78 (63, 89)^‡^ | 87 (81, 102)^‡^ | 133 (119, 161) | 118 (114, 130) |
| Ventilation [l∙min^-1^] | 70.2 (61.1, 75.1)^*^ | 87.5 (75.7, 107) | 92.9 (84.5, 107) | 131 (112, 162)* |
| Ventilation [l∙m^-2^ min^-1^] | 23.0 (21.1, 26.1)^†^ | 28.8 (26.6, 35.2) | 31.0 (28.2, 33.7) | 44.9 (39.2, 50.5)* |
| Breathing frequency [min^-1^] | 37.6 (31.0, 40.8) | 40.1 (32.2, 49.8) | 38.4 (36.9, 41.7) | 54.9 (47.5, 60.6)^†^ |
| Tidal volume [l] | 1.79 (1.50, 2.17) | 2.42 (2.16, 2.50) | 2.48 (2.16, 2.76) | 2.19 (2.07, 2.86) |
| Tidal volume [l∙m^-2^] | 0.63 (0.55, 0.67)* | 0.79 (0.69, 0.84) | 0.77 (0.71, 0.87) | 0.74 (0.71, 0.89) |
| P_ET_CO_2_ [mmHg] | 28.0 (26.5, 29.0)^‡^ | 27.0 (23.5, 28.0)^‡^ | 35.0 (32.5, 37.5) | 33.0 (29.5, 35.5) |
| Heart rate [bpm] | 100 (91, 133)^†^ | 126 (113, 140)* | 155 (145, 167) | 186 (183, 195)^†^ |
| RER | 1.11 (0.97, 1.15) | 1.16 (1.08, 1.23) | 1.16 (1.10, 1.23) | 1.18 (1.16, 1.25) |

The following indices mark Benjamin-Hochberg adjusted p-value of post hoc Kruskal Wallis tests against old control subjects: * p<0.05; ^†^ p<0.01; ^‡^ p<0.001. Bold values indicate p<0.05 for comparison of CCS with HF groups.

CHF, chronic heart failure; CCS, acute/chronic coronary syndrome; VT1, first ventilatory threshold; FVC, forced vital capacity; FEV_1_; forced expiratory volume in 1 s; V̇O_2_, oxygen uptake; P_ET_CO_2_, end-tidal carbon-dioxide partial pressure; RER, respiratory exchange ratio.
